# Cutaneous wound healing through paradoxical MAPK activation by BRAF inhibitors

**DOI:** 10.1038/ncomms12348

**Published:** 2016-08-01

**Authors:** Helena Escuin-Ordinas, Shuoran Li, Michael W. Xie, Lu Sun, Willy Hugo, Rong Rong Huang, Jing Jiao, Felipe Meira de-Faria, Susan Realegeno, Paige Krystofinski, Ariel Azhdam, Sara Marie D. Komenan, Mohammad Atefi, Begoña Comin-Anduix, Matteo Pellegrini, Alistair J. Cochran, Robert L. Modlin, Harvey R. Herschman, Roger S. Lo, William H. McBride, Tatiana Segura, Antoni Ribas

**Affiliations:** 1 Division of Hematology/Oncology, Department of Medicine, University of California, Los Angeles (UCLA), 14-635 Factor Bldg, 10833 Le Conte Avenue, Los Angeles, California 90095, USA; 2 Department of Chemical and Biomolecular Engineering, University of California, Los Angeles (UCLA), 7524 Boelter Hall, Los Angeles, California 90095, USA; 3 Division of Experimental Radiation Oncology, Department of Radiation Oncology, University of California, Los Angeles (UCLA), B3-109 CHS, Los Angeles, California 90095, USA; 4 Division of Dermatology, Department of Medicine, University of California, Los Angeles (UCLA), 52-121 CHS and 13-942 Factor Bldg, Los Angeles, California 90095, USA; 5 Department of Pathology and Laboratory Medicine, University of California, Los Angeles (UCLA), 924 Westwood Blvd. 730 and 1P-162 CHS, Los Angeles, California 90095, USA; 6 Department of Molecular and Medical Pharmacology, University of California, Los Angeles (UCLA), 329 Boyer Hall, Los Angeles, California 90095, USA; 7 Department of Biological Chemistry, University of California, Los Angeles (UCLA), 341 Boyer Hall, Los Angeles, California 90095, USA; 8 Department of Structural and Functional Biology, Institute of Biology, University of Campinas – UNICAMP, Campinas, 13083-970 Sao Paulo, Brazil; 9 Department of Microbiology, Immunology & Molecular Genetics, University of California, Los Angeles (UCLA), 52-121 CHS, Los Angeles, California 90095, USA; 10 Division of Surgical Oncology, Department of Surgery, 11-234 and 9-954 Factor Bldg, Los Angeles, California 90095, USA; 11 Jonsson Comprehensive Cancer Center, 10833 Le Conte Ave, Los Angeles, California 90024, USA; 12 Department of Molecular, Cell, and Developmental Biology, University of California, Los Angeles (UCLA), 3000C Terasaki Life Sciences Bldg, Los Angeles, California 90095, USA

## Abstract

BRAF inhibitors are highly effective therapies for the treatment of *BRAF*^*V600*^-mutated melanoma, with the main toxicity being a variety of hyperproliferative skin conditions due to paradoxical activation of the mitogen-activated protein kinase (MAPK) pathway in *BRAF* wild-type cells. Most of these hyperproliferative skin changes improve when a MEK inhibitor is co-administered, as it blocks paradoxical MAPK activation. Here we show how the BRAF inhibitor vemurafenib accelerates skin wound healing by inducing the proliferation and migration of human keratinocytes through extracellular signal-regulated kinase (ERK) phosphorylation and cell cycle progression. Topical treatment with vemurafenib in two wound-healing mice models accelerates cutaneous wound healing through paradoxical MAPK activation; addition of a mitogen-activated protein kinase kinase (MEK) inhibitor reverses the benefit of vemurafenib-accelerated wound healing. The same dosing regimen of topical BRAF inhibitor does not increase the incidence of cutaneous squamous cell carcinomas in mice. Therefore, topical BRAF inhibitors may have clinical applications in accelerating the healing of skin wounds.

## Abstract (web summary)

BRAF inhibitors often show skin-hyperproliferative side effects in melanoma patients. Here, the authors demonstrate that BRAF inhibitors can be used to enhance skin wound healing through the MAPK- ERK pathway activation that positively regulates the proliferation of keratinocytes.

## Graphical abstract

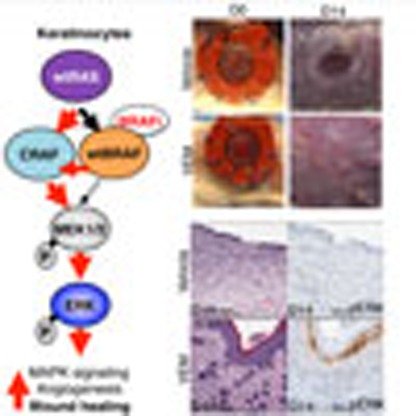

Paradoxical MAPK activation has long been recognized as a property of BRAF inhibitors^1^, where the preferential binding of the BRAF inhibitor to a BRAF protomer results in transactivation of its CRAF heterodimer partner. In the setting of strong upstream RAS-GTP signalling, membrane-localized RAF complexes then drive MEK/ERK activation, increased MAPK transcriptional output and enhanced cell proliferation (representative schematic of paradoxical MAPK activation in Supplementary Fig. 1)^2^,^3^,^4^,^5^.

In this study we show how we can take advantage of the mechanistic understanding of the skin hyperproliferative side effects of BRAF inhibitors to accelerate skin wound healing by inducing paradoxical MAPK activation in keratinocytes, which are the predominant cells in the epidermis. Keratinocytes play a major role in the initial proliferation stage of wound healing, restoring the barrier function of the epithelium^6^,^7^. We hypothesized that paradoxical MAPK activation induced by a BRAF inhibitor in these BRAF wild-type cells would accelerate cutaneous wound healing.

In our studies we demonstrate that vemurafenib promotes both proliferative and migratory effects on adult human epithelial keratinocytes (HEKa) *in vitro* by inducing phosphorylation of pErk and activation of the proliferative marker Ki67. Adding trametinib, a MEK inhibitor, to the vemurafenib-treated cultures, offsets proliferation and migration. Topical application of vemurafenib accelerates the healing of skin wounds in two cutaneous wound-healing murine models without promoting skin carcinogenesis. Consequently, topical BRAF inhibitors may be used to improve the healing of acute skin wounds.

## Results

### Vemurafenib effects on keratinocytes

We tested the effects of vemurafenib on HEKa cultured as a monolayer and subjected to a scratch, after which proliferating keratinocytes will grow to cover and heal the scratch. Replicate cultures with or without vemurafenib were placed in an incubator with an automated microscope analyzer to record the number of nucleated cells populating the original scratch region over time. Vemurafenib enhanced the covering of the scratch at 6, 8 and 12 h (Supplementary Fig. 2 and Supplementary Movies 1 and 2). Vemurafenib induced both proliferative and migratory effects on HEKa cells *in vitro* as combination cultures containing mitomycin C, a mitosis inhibitor, or NSC 295642, an inhibitor of cell motility, abolished both effects of vemurafenib using an assay in which migration and growth were initiated by removal of a central space sealant (Supplementary Fig. 3a). The enhanced proliferation and migration was inhibited by adding trametinib, a MEK inhibitor, to the vemurafenib-treated cultures (Supplementary Fig. 3b).

Three-dimensional soft agar assays showed proliferation of HEKa colonies upon exposure to vemurafenib, while the *BRAF*^*V600E*^ mutant melanoma line M249 had the expected opposite effect of decrease in colonies (Supplementary Fig. 4a,b). HEKa colonies not only increased in number but their mean spot size also increased significantly (*P*=0.007 by *t*-test, Supplementary Fig. 4c). Addition of trametinib decreased the number and size of HEKa colonies induced by vemurafenib (Supplementary Fig. 5).

**Figure 1:**
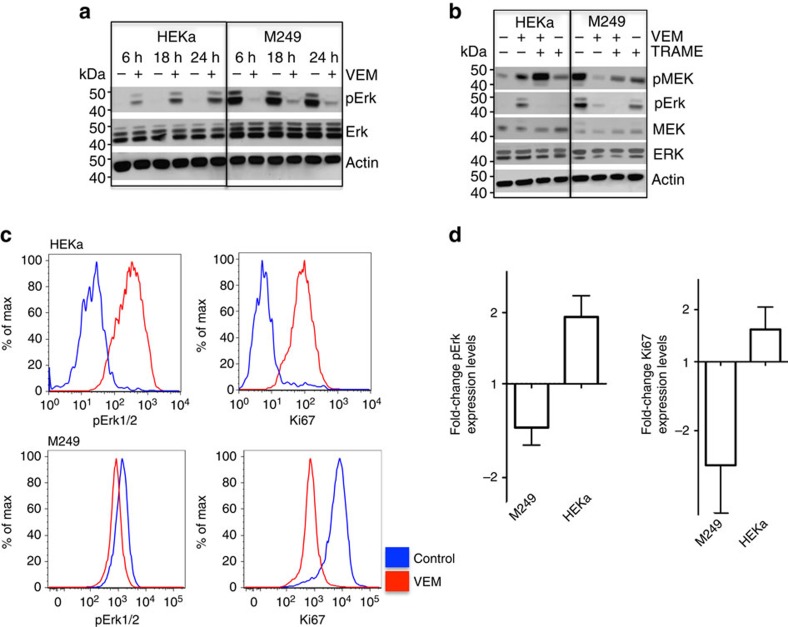
BRAF inhibition induces paradoxical MAPK activation in keratinocytes (HEKa). (**a**) Western blot analyses of pERK in HEKa compared with the *BRAF*^*V600E*^ mutant melanoma cell line M249 when treated with vemurafenib. (**b**) Levels of pERK and pMEK in HEKa compared with the *BRAF*^*V600E*^ mutant melanoma line M249 when treated with vemurafenib (VEM), trametinib (TRAME) or the combination for 24 h. (**c**) Histograms of intracellular flow cytometry analyses of HEKa and M249 cells treated with vehicle or vemurafenib (1.5 μM) and stained with pERK and Ki67 (staining controls represented in Supplementary Fig. 6). (**d**) Quantification of fold-change of pERK and Ki67 levels in three replicate cultures of HEKa and M249 cells treated with vemurafenib compared to vehicle. Error bars, mean±s.d.; *n*=3. Results are representative of two experiments.

In HEKa and human *BRAF*^*V600E*^ melanoma cultures, we analysed MAPK signalling by western blot (Fig. 1a,b), as well as pERK and cell proliferation by quantitative intracellular flow cytometry (Fig. 1c,d and Supplementary Fig. 6). Vemurafenib decreased pERK in the *BRAF*^*V600E*^ mutant human melanoma cell line M249, as expected, whereas it induced a paradoxical increase in pERK in HEKa cells. Furthermore, in the presence of vemurafenib, the proliferative marker Ki67 decreased in M249 melanoma cells while it increased in HEKa cells (Fig. 1c,d).

### Vemurafenib enhances and trametinib inhibits wound healing

**Figure 2:**
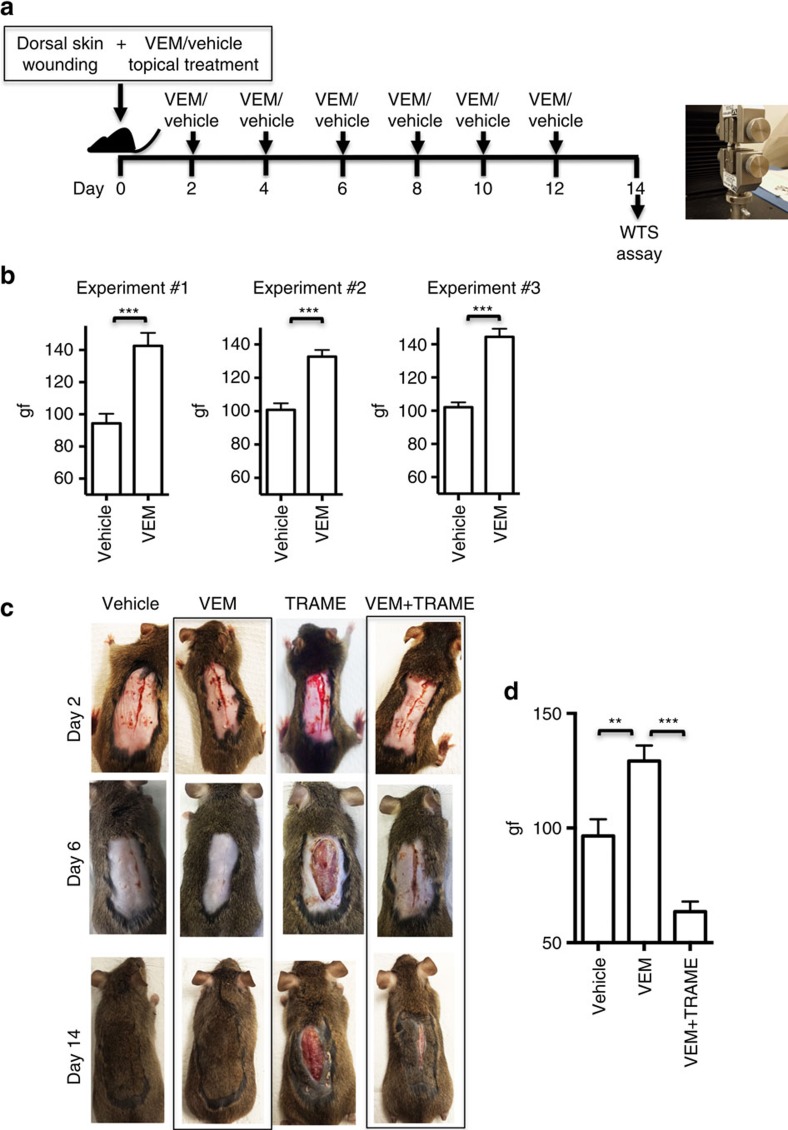
BRAF inhibition accelerates and MEK inhibition suppresses wound healing. (**a**) Schematic representation of the wound-healing studies performed in C3H mice. (**b**) Wound tensile strength (WTS) using 2 mm strips of wound-containing skin in three replicate experiments, each with eight mice per group. WTS is represented as gram force (gf) per 2 mm strip (*P*<0.0001). (**c**) Representative images of mice treated topically with vehicle, vemurafenib (VEM, 2 mg), trametinib (TRAME, 0.2 mg) and the combination of vemurafenib and trametinib (VEM+TRAME) on days 2, 6 and 14. (**d**) WTS in 8 mice per group, as gram force (gf) comparing vehicle, vemurafenib and the combination of vemurafenib and trametinib (*P*<0.0001). Error bars in **b** and **d**, mean±s.d.; *n*=8. Every graph represents one experiment with 8 replicates.

We used two cutaneous wound-healing murine models to test the effects of vemurafenib to improve the healing of skin wounds. In an incisional wound-healing mice model (Fig. 2a), vemurafenib or vehicle control was applied topically every other day for seven doses in eight mice per group. On day 14, the gain in wound tensile strength (WTS, in gram force per 2 mm (gf per 2 mm)) was analysed using a tensiometer. In three independent replicate experiments (eight mice per group in each experiment; seven strips per wound), vemurafenib-treated mice significantly improved WTS compared with vehicle-treated controls (52%, 33% and 42%, respectively; *P*<0.0001 by *t*-test for all three experiments; Fig. 2b). The administration of trametinib in addition to vemurafenib reduced the WTS by 51% compared with when using vemurafenib alone (*P*<0.0001 by *t*-test). Trametinib on its own inhibited wound healing to a point where the skin wound closure was too compromised and the WTS could not be assessed (Fig. 2c,d). The area of the wounds and their surroundings were analysed histologically by two pathologists blinded to the study groups (RRH and AJC). Vemurafenib-treated wounds displayed an accelerated proliferative stage of wound healing as evidenced by quantitating the extent of epidermal hyperplasia on both sides of the healing wounds. No re-epithelialization was observed in the trametinib alone and vemurafenib plus trametinib groups at this time point (Supplementary Fig. 7).

### Vemurafenib enhances re-epithelialization

**Figure 3:**
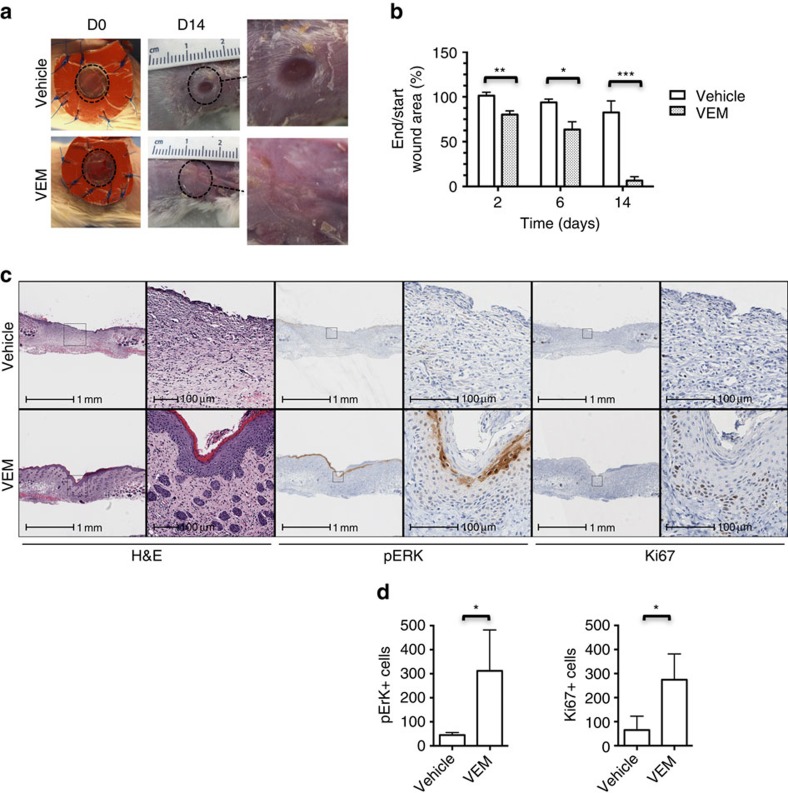
BRAF inhibition accelerates wound healing in an excisional wound model. (**a**) Representative images of vehicle-treated and vemurafenib (VEM)-treated mice on day 0 and day 14. Original wound area is marked with dotted circles and amplified on the right side. (**b**) Percentage of wound closure on day 2, 6 and 14. (**P=0.004 (*n*=6), *P=0.02 (*n*=4; two mice died due to haemorrhagic wounds), ***P=0.0002 (*n*=6)). (**c**) Representative photomicrograph hematoxylin and eosin (H&E), pERK and Ki67 staining images in the presence and absence of vemurafenib by day 14. (**d**) Bar graph representing the quantification of pERK+ and Ki67+ cells in the vehicle- and vemurafenib-treated wounds on day 14 (*P=0.02; *n*=4). Error bars in **b** and **d**, mean±s.d. Bar graphs represent one experiment with 4 to 6 replicates per group.

In an excisional wound-splinting model in Balb/c mice^8^, we induced 6-mm round wounds on the back of mice; splinting rings were tightly adhered and sutured to the skin around the wounds, preventing wound closure caused by skin contraction (representative images in Supplementary Fig. 8). Vemurafenib (2 mg) or DMSO was applied on the wounds on days 0, 2 and 4, and per cent wound closure was sequentially measured. The wounds treated with vemurafenib showed a significantly higher percentage of wound closure compared with the ones treated with vehicle on days 2, 6 and 14 (*P*=0.004, *n*=6; *P*=0.02, *n*=4; *P*=0.0002, *n*=6, respectively, by *t*-test; Fig. 3a,b). The area of the wounds and their surroundings were analysed histologically and pERK+ and Ki67+ cells were quantified using digital pathology (Fig. 3c,d). Compared with controls, the healing process in the presence of vemurafenib was accelerated showing re-epithelialization by day 6 (Supplementary Fig. 9). The skin surface integrity was re-established by day 14 in the vemurafenib-treated wounds, whereas remodelling and dermal reparative fibrosis was delayed in the control group (Fig. 3c). The number of pERK+ and Ki67+ cells in the wounds treated with vemurafenib was statistically significantly higher by day 14 compared with the control group (*P*=0.02 by *t*-test; *n*=4; Fig. 3c,d), demonstrating paradoxical MAPK leading to enhanced epithelial cell proliferation.

### Vemurafenib upregulates MAPK and wound-healing-related signatures

**Figure 4:**
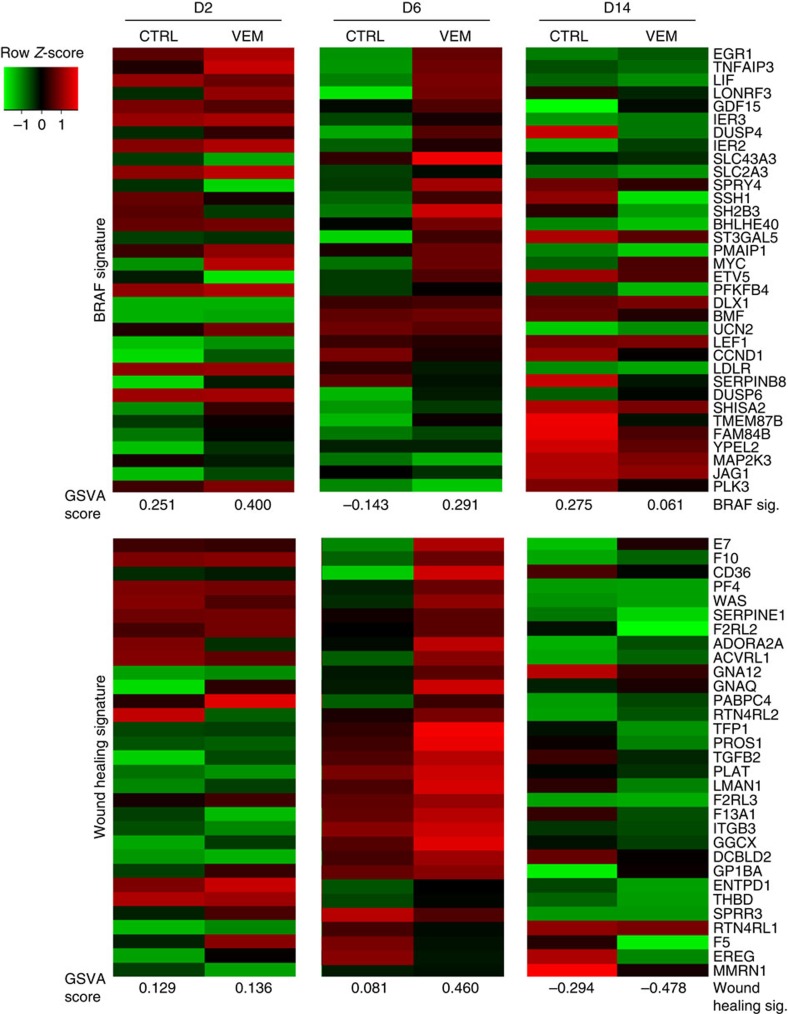
Gene expression profiling of cutaneous wound healing in mice. Full-thickness skin resected around the areas of the initial wound was analysed for gene expression profiling by RNASeq. Top, Expression, regulation and overall enrichment of BRAF signature genes as measurement of MAPK pathway activity. Bottom, expression changes and enrichment of wound-healing signature genes (GO: 0042060). There is enrichment of the BRAF signature at day 2 and 6 in vemurafenib-treated wounds, with a significant increase of the wound-healing signature at day 6. Enrichment scores, GSVA (the scores are normally distributed around 0 where negative values indicate relative de-enrichment of the signature and positive values indicate enrichment).

To further characterize epithelial skin repair, the skin samples obtained from the incisional wound-healing model were analysed by RNASeq. The list of differentially expressed genes on day 2, 6 and 14 (Supplementary Data 1) was compared with published data for the transcripts that were differentially modulated by blocking oncogenic MAPK signalling downstream of mutated BRAF^*V600E*^ using BRAF inhibitors^9^, and to genes within the gene ontology term ‘wound healing' (GO:0042060)^10^. By day 6, both signatures were enriched significantly in the vemurafenib-treated samples compared to their respective controls (overall enrichment scores were computed based on Single-sample Gene Set Variation Analysis (GSVA)^11^ (Fig. 4). A more pronounced decrease of both the BRAF^*V600E*^ and wound-healing signatures was observed in the vemurafenib-treated wounds by day 14, consistent with a more rapid healing. Additionally, our gene output was compared with an early stage wound-healing signature, with mostly pro-inflammatory genes involved in the first stages of wound healing^12^ and a post-operatory wound-healing signature^13^. These two wound-healing signatures were also enriched by day 6 in the vemurafenib-treated groups as opposed to the control group (Supplementary Fig. 10 and Supplementary Data 2). Genes recognized to be associated with wound healing^14^ were upregulated in vemurafenib-accelerated wound-healing wounds compared with control wounds (Fig. 4 and Supplementary Fig. 10).

### Transcriptional signature in vemurafenib-treated wounds

**Figure 5:**
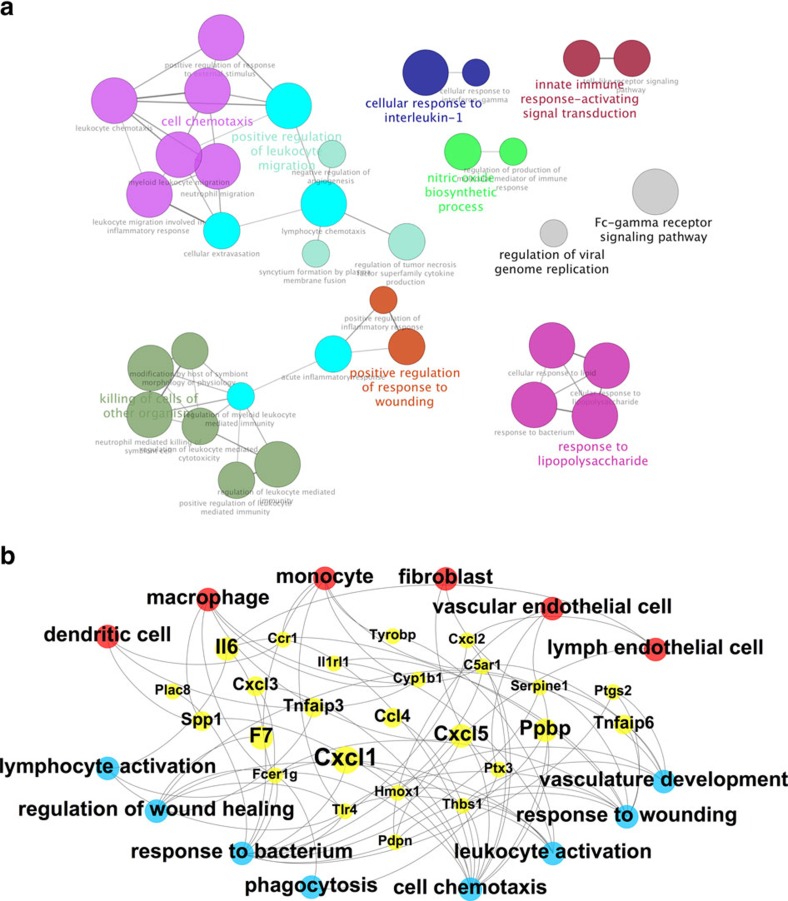
Effect of vemurafenib on the biological pathways involved in wound healing. (**a**) Significantly activated biological process/pathways by day 6 of vemurafenib treatment based on the enriched gene ontology (GO) clusters visualized by ClueGO. (**b**) Integrated view highlighting specific wound-healing cell subsets (red; with signature enrichments), their upregulated genes (yellow) and enriched wound-healing-related processes (blue) in the transcriptome of mice wounds treated with vemurafenib by day 6. Gene node size represents induction at day 6, with the largest node representing a log2 (FC) of 4.54 and smallest node representing a log2 (FC) of 1.03.

Enrichment of signatures for dendritic cells, macrophages, monocytes, fibroblasts and vascular and lymphatic endothelial cells was observed in the wounds treated with vemurafenib as compared with the control wounds (Supplementary Fig. 11). We confirmed the increase in macrophages with topical vemurafenib by IHC analysis (Supplementary Fig. 12). As expected, the increase in macrophages was abolished with the co-administration of trametinib. To elaborate on the activated pathways associated with the increased presence of wound-healing cell subsets at this time point, we analysed for enriched gene ontology (GO) biological processes on the genes up-expressed in the vemurafenib-treated wounds (⩾2-fold increase) using ClueGo^15^. We found clear enrichment of pathways involved in lymphocyte activation, vascular development and response to wounding (Fig. 5a). Integration of the enrichments of cell subset signatures, gene and pathway upregulation showed enhanced recruitment of wound-healing-specific cell subsets which results in stronger activation of the inflammatory and angiogenic wound-healing processes (Fig. 5b). To confirm these findings, the levels of COX-2+ and IL-6+ cells were quantified by immunohistochemistry. The wounds treated with vemurafenib had higher IL-6+ and COX-2+ cells at the proliferation stage, on day 6 (Supplementary Fig. 13), which is consistent with our integrated RNAseq data shown in Fig. 5b. As a further verification of our RNAseq data, RT-PCR for Egr-1, TNFAIP3 and F7, for total skin wounds, was performed. These three genes are upregulated in our gene signature (Fig. 5b) and our results on the RT-PCR verified this increase in vemurafenib-treated wounds by day 6 post treatment (Supplementary Fig. 14).

### Enhanced angiogenesis with vemurafenib

**Figure 6:**
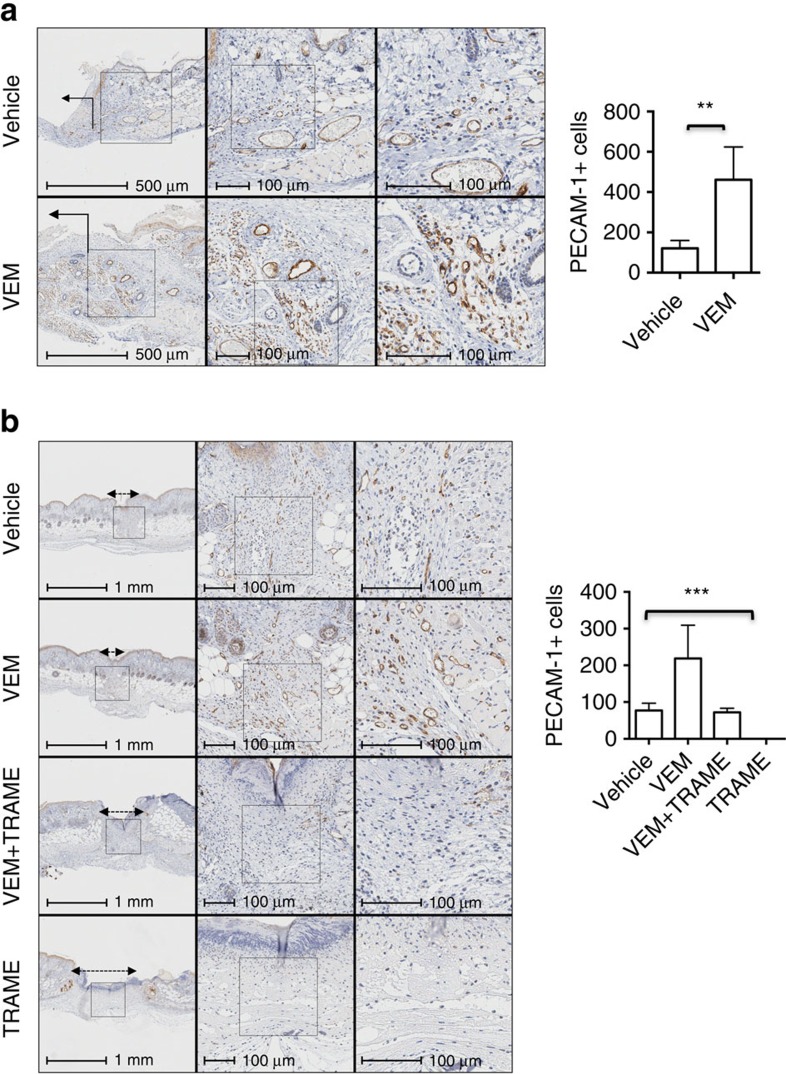
Angiogenesis is increased in mice wounds treated with vemurafenib. (**a**) Representative photomicrograph immunohistochemistry images of PECAM-1+ cells in an excisional wound splinting model of mice treated with vehicle or vemurafenib on day 6 post-treatment and bar graph, on the right side, representing the quantification of PECAM-1+ cells on the vehicle- and vemurafenib-treated wounds on day 6 (*P*=0.006 by *t*-test; *n*=4). Wound area indicated with an arrow. (**b**) Representative photomicrograph immunohistochemistry images of PECAM-1+ cells in an incisional wound model of mice treated with vehicle, vemurafenib (VEM), trametinib (TRAME) or the combination (VEM+TRAME); (*P*=0.0002 by one-way ANOVA). Trametinib alone completely depleted the number of PECAM-1+ cells. Wound area between double head arrows. Error bars, mean±s.d. Bar graphs represent one experiment with 4 replicates per group.

To confirm the beneficial effects of vemurafenib treatment on angiogenesis, we quantified PECAM-1+ cells in the excisional and incisional wound areas. In the excisional wound model there was a 74% increase in the number of PECAM-1+ cells in vemurafenib-treated wounds compared with the control wounds at day 6 post-treatment (*P*=0.006 by *t*-test *n*=4; Fig. 6a). A 65% increase of PECAM-1+ cells was observed in the incisional wound model in wounds treated with vemurafenib (*P*=0.02 by *t*-test), consistent with the enrichment of endothelial cells observed in the RNASeq data (Fig. 6b). This increase was reversed by the addition of topical trametinib (0.2 mg), with a 67% decrease in the number of PECAM-1+ cells at day 6 (*P*=0.01 by *t*-test; vemurafenib versus vemurafenib+trametinib). Trametinib alone completely depleted PECAM-1+ cells (Fig. 6b).

### Vemurafenib does not induce skin epidermal tumours

**Figure 7:**
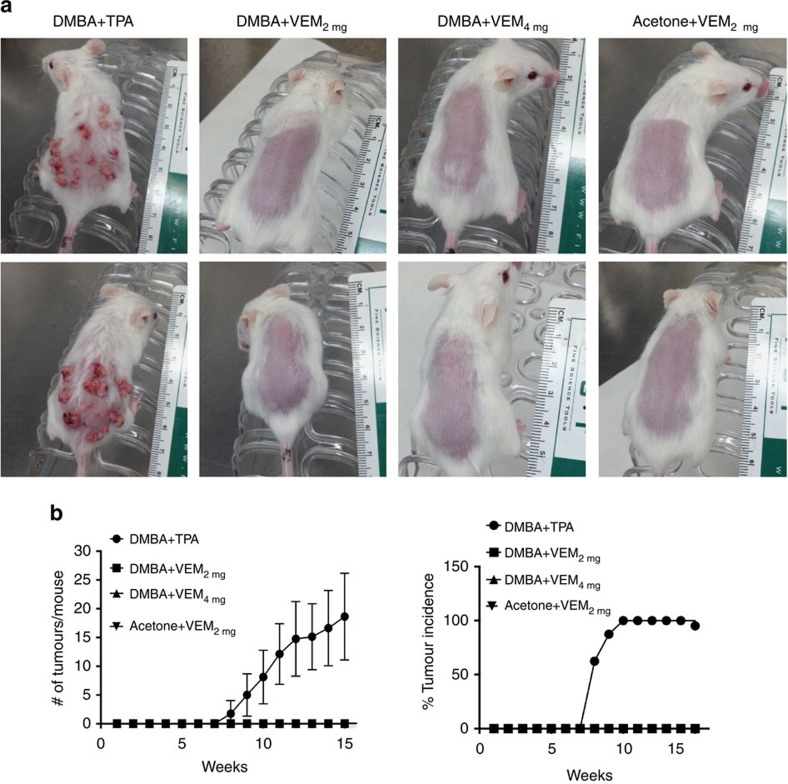
Vemurafenib does not induce skin tumours when applied topically to mice. (**a**) Representative images of mice from each study group on week 15 of treatment. Topical application of 7,12-Dimethylbenz[a]anthracene (DMBA) to FvB/N mice followed by 12-O-tetradecanoylphorbol-13-acetate (TPA) induced skin papillomas and squamous cell carcinomas by week 8 of treatment. In the control (DMBA+TPA) group all eight mice developed tumours. In the groups with DMBA or acetone control, followed by topical application of vemurafenib (VEM, 2 or 4 mg per mice) no skin tumours were induced. (**b**) Graph representations of tumour count and percentage of tumour incidence per week. Error bars in **b**, mean±s.d.; *n*=8.

To analyse the possibility of a cutaneous tumour-promoting activity of topically applied vemurafenib, we used the well-established two-stage skin carcinogenesis model^16^,^17^. Topical application of DMBA or acetone was followed either with topical TPA as a positive control for skin carcinogenesis or with topical vemurafenib, twice a week for 15 weeks^16^. The only mice that developed skin carcinogenesis were the group treated with DMBA plus TPA (Fig. 7). No skin papillomas, keratoacanthomas or squamous cell carcinomas occurred in mice treated with DMBA plus vemurafenib at the concentrations that enhanced wound healing (2 mg) or higher (4 mg). Therefore, topical vemurafenib did not promote skin carcinogenesis even after mice were exposed to an initiating carcinogen application that results in the presence of epithelial cells bearing *RAS* mutations^16^,^18^.

## Discussion

In the current work, we tested whether the skin hyperproliferative side effects of BRAF inhibitors can be exploited to accelerate skin wound healing by inducing paradoxical MAPK activation. These findings show how the BRAF inhibitor vemurafenib enhances proliferation and migration of keratinocytes *in vitro* and in two *in vivo* mice models, when applied topically on the skin wounds of mice.

Collectively, these studies demonstrate that the phenomenon of paradoxical MAPK activation by BRAF inhibitors may be exploited to enhance skin wound healing. Topical application of a BRAF inhibitor resulted in the accelerated healing of skin wounds by primarily acting on the proliferative stage of wound healing, with improvement in multiple other events in wound healing including inflammation and angiogenesis. These benefits were offset by adding a MEK inhibitor that is known to inhibit paradoxical MAPK activation^18^, while this topical therapy did not result in tumour promotion. Therefore, topical BRAF Inhibitors could be used to accelerate the healing of acute skin wounds, such as abrasions and surgical incisions.

## Methods

### Data reporting

No statistical methods were used to predetermine sample size. The experiments were not randomized. The investigators were not blinded to allocation during experiments and outcome assessment.

### Cell proliferation and migration assays

HEKa were purchased from Invitrogen (Grand Island, NY; C-005-5C). HEKa cells (25,000 per well) were plated on 96-well ImageLock cell migration plates (Essen Bioscience, Ann Arbor, MI, USA) and incubated overnight. Then, the cell monolayer was scratched with a 96-pin WoundMaker (Essen Bioscience), and the cells washed with PBS (phosphate-buffered saline) before adding cell medium. Cells were maintained in culture with a concentration of 1.5 μM of vemurafenib until complete scratch closure. Cell migration was monitored by a microscope gantry inside a cell incubator, which was connected to a networker external controller hard drive that gathered and processed image data (Incucyte, Essen Bioscience, Ann Arbor, MI, USA). This allows an automated and non-invasive method of monitoring live cells in culture. HEKa and M249 cells were plated on OrisTM cell migration plates (Platypus Technologies, Madison, WI, USA), treated with vehicle, vemurafenib (1.5 μM), trametinib (1 μM), mitomycin C (10 μg ml^−1^), NSC 295642 (1 μg ml^−1^) or combination, and loaded with CellTrackerTM Green CMFDA (5-chloromethylfluorescein diacetate) probe (Life Technologies, Carlsbad, CA, USA). Mitomycin C and NSC 295642 were purchase from Sigma-Aldrich, Sant Louis, MO, USA. Cell migration was assessed using a BioSpot Series 5 UV analyzer (Cellular Technology Limited, Cleveland, OH, USA).

### Colony-forming assays

Twenty-four-well plates were covered with 300 μl of serum-free RPMI 1640 (Fisher Scientific, Hampton, NH, USA) with 0.6% Noble agar (BD Biosciences, San Jose, CA, USA), and incubated at 37 °C overnight, until solid. Three-hundred microlitres of a suspension of HEKa and M249 cells (15,000 cells per ml) in a 1:1 mixture of growth medium with a concentration of 1.5 μM of vemurafenib and growth factor-reduced matrigel (BD Biosciences) were added to each well. After 1 week, automated colony quantification was performed using a BioSpot Series 5 UV analyzer.

### Intracellular flow cytometry analysis

HEKa and M249 cells treated with a concentration of 1.5 μM of vemurafenib or vehicle control were fixed with formaldehyde to a final concentration of 1.6%, and permeabilized using methanol (90%). Then, they were washed in staining buffer (sterile PBS, 0.5% BSA, 0.01% sodium azide, NaN_3_), and stained with Alexa Flour 647 mouse anti-ERK1/2 (pT202/pY204) antibody and PE mouse anti-human Ki67 antibody (BD Pharmingen, San Jose, CA, USA). After incubation, cells were washed again and resuspended in 3 ml of staining buffer. A total of 30,000 cellular events were acquired for analysis. Data was analysed using FlowJo (Tree Star Inc., Ashland, OR, USA). HEKa and M249 cells were routinely tested for mycoplasma and were found to be negative.

### Western blotting

M249 and HEKa cells were treated in duplicate with a concentration of 1.5 μM of vemurafenib or vehicle control. Primary antibodies included p-ERK Thr204/205 (1:1,000, #9101), ERK (1:1,000, #4094), Ki67 (1:1,000, #9449) and beta-actin (1:3,500, #8457) (all from Cell Signalling Technology, Danvers, MA, USA). Immuno-reactivity was revealed with an ECL-Plus kit, using a Typhoon scanner (both from Amersham Biosciences Co, Piscataway, NJ, USA)^17^. Uncropped scans of the most important immunoblots are shown in Supplementary Fig. 15.

### Incisional dorsal wound model

C3Hf/Kam (H2-k) female mice were used for wound-healing studies at 8–10 weeks of age. They were bred at UCLA and used under the Animal Research Council (ARC) protocol # 2013-066-01 ‘Wound-Healing with BRAF inhibitors'. Full-thickness wounds ∼2.5 cm long were made in the shaved dorsal skin of anaesthetized mice as described^19^, with ketamine/xylazine as an anaesthetic. Clinical grade vemurafenib pills (Zelboraf, Genentech, South San Francisco, CA, USA) were grinded and dissolved in dimethylsulfoxide (DMSO; Fisher Scientific) and phosphate buffered saline (PBS; 1:4) to a concentration of 40 μg μl^−1^ and 50 μl of the mixture (or DMSO in PBS as vehicle control) was added topically. Wounds were closed with 3–4 clips, which were removed after 2 days. Vemurafenib suspension (2 mg) or vehicle control was re-applied topically to the wound site on days 2, 4, 6, 8, 10 and 12. Two weeks after wounding, a square of skin containing the wound was removed from euthanized mice and cut into seven 2-mm strips of 20 mm in length with a multiblade device, so that each 2-mm-wide strip contained a horizontal wound sample^20^. The strips were spread on filter papers soaked in ice-cold PBS in covered Petri dishes until WTS measurement, as previously described^21^ using an Instron tensiometer (Model 3342; Instron, Norwood, MA, USA). The skin strips were stretched at a rate of 1 cm min^−1^ to breaking point to obtain the peak WTS in gf per 2 mm. The WTS of unwounded skin from 12 weeks old mice is 250 gf (ref. 20).

### Excisional skin wound splinting model

Seven- to nine-week old female Balb/c mice were used for these studies under the ACR protocol #2010-011-13F. Mice were anaesthetized with 2–3% isoflurane in an induction chamber and kept under anaesthesia during the whole surgery. The back of the mice was shaved, washed with betadine and 70% ethanol and a dose of buprenorphine (2.5 mg kg^−1^) was administered, subcutaneously, before the surgery. Two excisional wounds were made in the skin aside the midline of the animal using a 6-mm biopsy punch. Twenty microliter of vemurafenib (0.1 mg μl^−1^) or DMSO was applied topically on the wounds 1 min before suturing of the splinting rings. The splinting rings have an 8-mm transparent window, which was covered with Tegaderm to allow visualization and measurement of the wound size. All animals were observed daily for signs of inflammation and pain for the first 48 h post surgery. Vemurafenib or DMSO was repeatedly applied on day 2 and 4. Wounds were photographed at day 0, day 2, day 6 and day 14, based on which the percentages of wound closure were calculated.

### Histological analyses

Dorsal skin wounds from CH3 mice treated with vemurafenib suspension or DMSO in saline-control suspension were excised at day 2, 6 and 14. They were fixed in 10% neutral buffered formalin and embedded in paraffin and stained with hematoxylin and eosin (H&E) using standard methods. Balb/c mice were sacrificed at day 2, 6 and 14 with isoflurane overdose. Two 8-mm round pieces of tissue were collected from each Balbc/c mouse containing the whole-wound area and the surrounding tissue and skin, cut precisely in half at the midline of the wound and fixed in 1% paraformaldehyde (PFA) for 16–18 h at 4 °C, dehydrated in 70% EtOH, and then paraffin embedded. Sections were cut at 4 μm, deparaffinized with xylene and descendant ethanol and then incubated in 3% H_2_O_2_ for 10 min. After a wash in distilled water, the slides were incubated for 25 min in citrate buffer pH6 (Invitrogen, Carlsbad, CA, USA) at 95 °C using a vegetable steamer. The slides were brought to room temperature, rinsed in PBST (PBS-containing 0.05% Tween-20), and then incubated at room temperature with 1:100 Ki67 antibody (DAKO, Carpinteria, CA, USA), 1:1,000 PECAM-1 antibody (Santa Cruz Biotechnology, Dallas, Texas) or 1:200 IL-6 antibody (Abcam, Cambridge, MA, USA) for 1 h and 1:10 phospho-ERK Ab (Cell Signaling Technology, Danvers, MA, USA), 1:1,500 CD68 antibody (Abcam, Cambridge, MA, USA) or 1:25 COX-2 antibody (Cayman Chemical, Ann Arbor, MI) overnight at 4 °C. The stained slides were rinsed with PBST and incubated at room temperature with 1:200 polyclonal Rabbit anti-rat immunoglobulin/Biotinylated Ab (Dako, Carpinteria, CA #E0468) for 30 min. All slides were rinsed with PBST, and incubated with Dako EnVision+ System –HRP Labelled Polymer Anti-Rabbit (Dako, Carpinteria, CA, USA) at room temperature for 30 min. After a rinse with PBST, the slides were incubated with DAB (3,3′-diaminobenzidine) for visualization. Subsequently, the slides were washed in tap water, counterstained with Harris' Hematoxylin, dehydrated in ethanol, and mounted with media. The imaging and quantification of our cell-based immunohistochemistry was performed with the HALO Next Generation Imaging analysis software (Indica Labs; Corrales, NM). HALO measures and reports individual cell data maintaining an interactive link between cell metrics and cell imagery. The number of pERK+ Ki67+ and PECAM-1+ cells was automatically counted with the HALO software. Three 20 × fields of view from each side of the wound were automatically counted for pERK and Ki67 stains. PECAM-1+ and CD68+ cells were automatically counted on each side of the wound edges where the granulation tissue starts (1mm length each side) on the excisional wound-splinting model, and in the entire wound area on the incisional wound model. IL-6+ and COX-2+ cells were automatically counted in the entire wound area on the incisional wound model.

### RNAseq analysis

RNA from mice skin samples in each treatment group were extracted (RNeasy Mini Kit, Qiagen, Valencia, CA, USA) on days 2, 6 and 14 and sent for RNAseq analysis using 2 × 100 bp paired end Illumina HiSeq2000 (Illumina, San Diego, CA, USA) sequencing run. Raw sequences were mapped to the mouse mm9 reference sequence by TOPHAT. The normalized gene expression levels of each were expressed in FPKM values as generated by the programme cuffquant and cuffnorm on TOPHAT's BAM output^19^. We applied the options ‘--frag-bias-correct', ‘--multi-read-correct' and ‘--compatible-hits-norm' on the cuffquant run. The heatmap of the MAPK and wound-healing signatures was generated based on the signature genes' row-normalized FPKM levels by the R package gplots. GSVA score was computed using normalized read counts as described^11^. Normalized read counts (computed by Cuffnorm on the RNAseq BAM files) of the mouse tissue with/without treatment of vemurafenib at day 2, 6 and 14 were supplied to the dermDB database^13^. Different immune cell-type gene sets that we used were reported, previously^22^. Enrichment scores were computed as the average difference of each gene in a gene set against its mean across all samples. Row-normalized enrichment scores of the immune gene sets were visualized and the *P*-values of the enrichment score differences were computed based on a null distribution generated by permutation of the gene labels (*n*=100,000). *P*-values were adjusted using Benjamini–Hochberg method. Specific biological processes on day 6 with vemurafenib treatment (with control day 6 as reference) were nominated using GO enrichment analysis on the upregulated genes (minimum 2-fold upregulation) in the treated group. The enriched GO terms were computed and visualized using ClueGO (ref. 15). The integrated panel highlighting relations among enriched genes, gene processes and specific immune subsets was created using the Gephi software^23^. The data was submitted to the GEO repository (accession number GSE74558).

### Quantitative PCR

q-PCR was performed using a one-step reverse transcription kit developed specifically for SYBR Green-based real-time PCR (*Power* SYBR Green RNA-to-CT 1-Step Kit, Thermo Fisher Scientific, Carlsbad, CA, USA), with a standard quantitation-comparative Ct procedure as set by the manufacturer. Triplicate reactions (25 μl) of each experimental sample were prepared with the following primers: TNFAIP3 (Fwd: 5′- CTGACCTGGTCCTGAGGAAG -3′; Rev: 5′- GCAAAGTCCTGTTTCCA -3′), F7 (Fwd: 5′ GACTTTGACGGTCGGAACTGTG 3′; Rev: 5′ GCGGCTGCTGGAGTTTCTTT 3′) and Egr-1 (Fwd: 5′- GACGAGTTATCCCAGCCAAA -3′; Rev: 5′- GGCAGAGGAAGACGATGAAG -3′). Data were normalized to *Bactin* levels.

### Carcinogenesis studies

Female FVB/N mice were purchased from Charles River Laboratory (Wilmington, MA, USA). Tumour induction procedures were carried out in accordance with ARC protocol #2013-066. The two-stage carcinogenesis procedures were performed as described previously^24^,^25^ with 8 mice per group. DMBA and TPA were purchased from Sigma. Clinical grade vemurafenib pills were grinded and dissolved in DMSO; to a concentration of 0.02 and 0.04 mg μl^−1^ and 100 μl of the mixtures (or DMSO as vehicle control) was added topically on the back of the mice. Vemurafenib suspension (2 or 4 mg) or vehicle control was re-applied topically to the back of the mice twice a week for 15 weeks.

### Statistical analysis

Data were analysed with GraphPad Prism (version 5) software (GraphPad Software, La Jolla, CA, USA). Significance was determined by unpaired two-tailed Student's *t*-test or one-way analysis of variance (ANOVA). Variance was similar between the groups that were statistically compared.

### Data availability

All relevant data are included with the manuscript as figure source data or Supplementary Information Files. The RNAseq data can be accessed at the GEO repository (accession number GSE74558).

## Additional information

**How to cite this article:** Escuin-Ordinas, H. *et al*. Cutaneous wound healing through paradoxical MAPK activation by BRAF inhibitors *Nat. Commun.* 7:12348 doi: 10.1038/ncomms12348 (2016).

## Supplementary Material

Supplementary Figures Supplementary Figures 1-15

Supplementary Movie 1 Video capture of the cell proliferation scratch assay of human epithelial adult keratinocytes (HEKa) in saline and DMSO (without vemurafenib).

Supplementary Movie 2 Video capture of the cell proliferation scratch assay of human epithelial adult keratinocytes (HEKa) in the presence of vemurafenib.

Supplementary Data 1 List of up-regulated and down-regulated genes in the wounds treated with vemurafenib at day 2, 6 and 14. The values listed are log2 transformed after adding a pseudo FPKM value of 0.1 to remove large fold changes caused by low FPKM values (>0.1). Supplementary Data 2. List of genes involved in the postop and early wound healing signatures.

Supplementary Data 2 List of genes involved in the postop and early wound healing signatures.
