# Supplementary material for: Cutaneous wound healing through paradoxical MAPK activation by BRAF inhibitors
Source: Nat Commun. 2016 Aug 1;7:12348. doi: 10.1038/ncomms12348 (PMC4974650; doi:10.1038/ncomms12348)
Supplement: Supplementary Figures — 1-15 [file ncomms12348-s1.pdf]

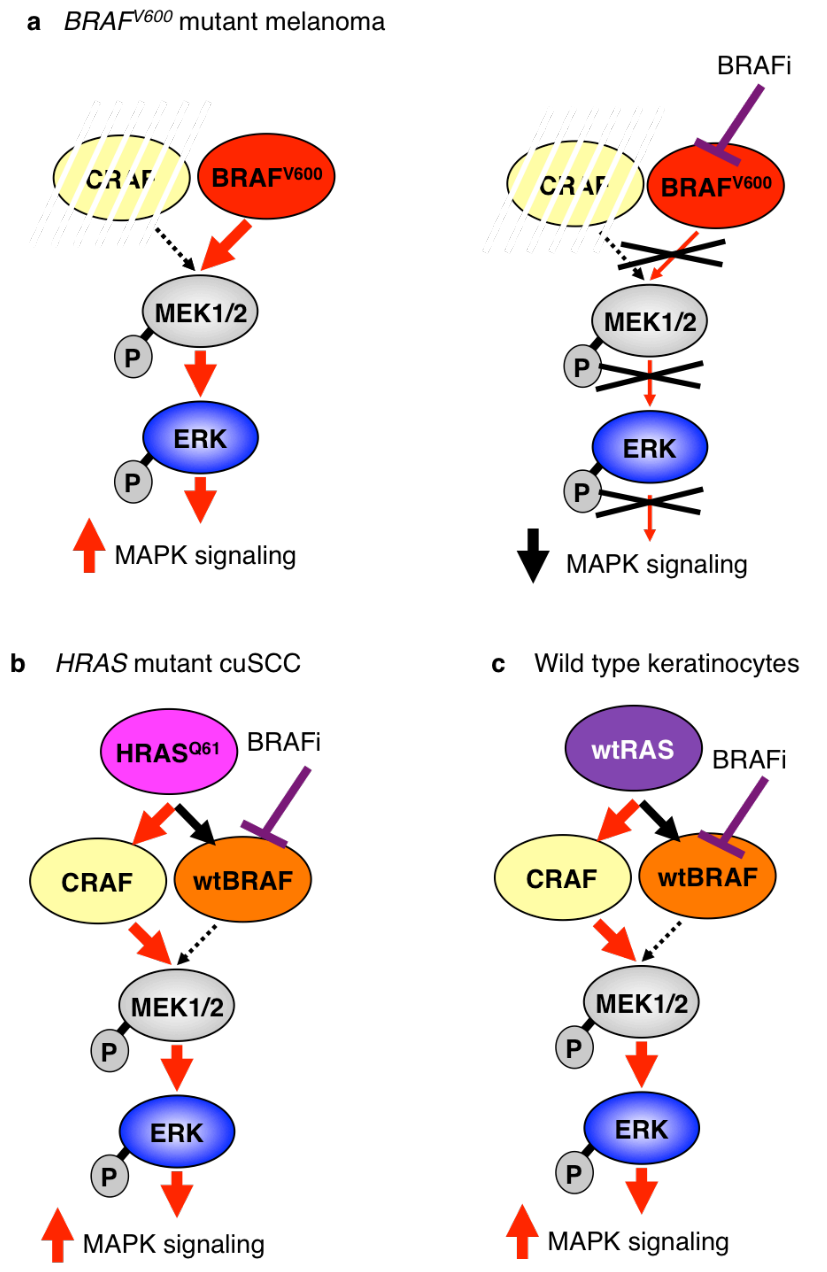

Supplementary Figure 1. Schematic of BRAF inhibitor effects in *BRAF*^{V600} mutated melanoma and paradoxical MAPK activation in *BRAF* wild type cells. **a**, In *BRAF*^{V600} mutated melanoma, oncogenic BRAF results in constitutive MAPK signaling without requiring the contribution of CRAF (left panel). When these cells are treated with a BRAF inhibitor

(BRAFi), then MAPK pathway signaling is inhibited. **b**, In *HRAS* mutated cutaneous squamous cell carcinomas (cuSCC), RAS is constitutively activated and brings together wild type BRAF and CRAF. The BRAF inhibitor shows preferential binding to the BRAF protomer, which results in transactivation of its CRAF heterodimer partner and drives MEK/ERK activation, MAPK transcriptional output, and enhanced cell proliferation. This is known as paradoxical MAPK activation. **c**, In keratinocytes that have non-mutated (wild type) RAS and BRAF, when cultured *in vitro* and in healing wounds exposure to the BRAF inhibitor induces paradoxical MAPK activation and increased cell proliferation. Not depicted in this schematic is the effect of adding a MEK inhibitor, which would be anticipated to block paradoxical MAPK activation in keratinocytes and result in decreased MAPK signaling (decreased pERK), leading to decreased cell proliferation (decreased Ki67), ultimately resulting in inhibiting the beneficial effects of the BRAF inhibitor to improve wound healing.

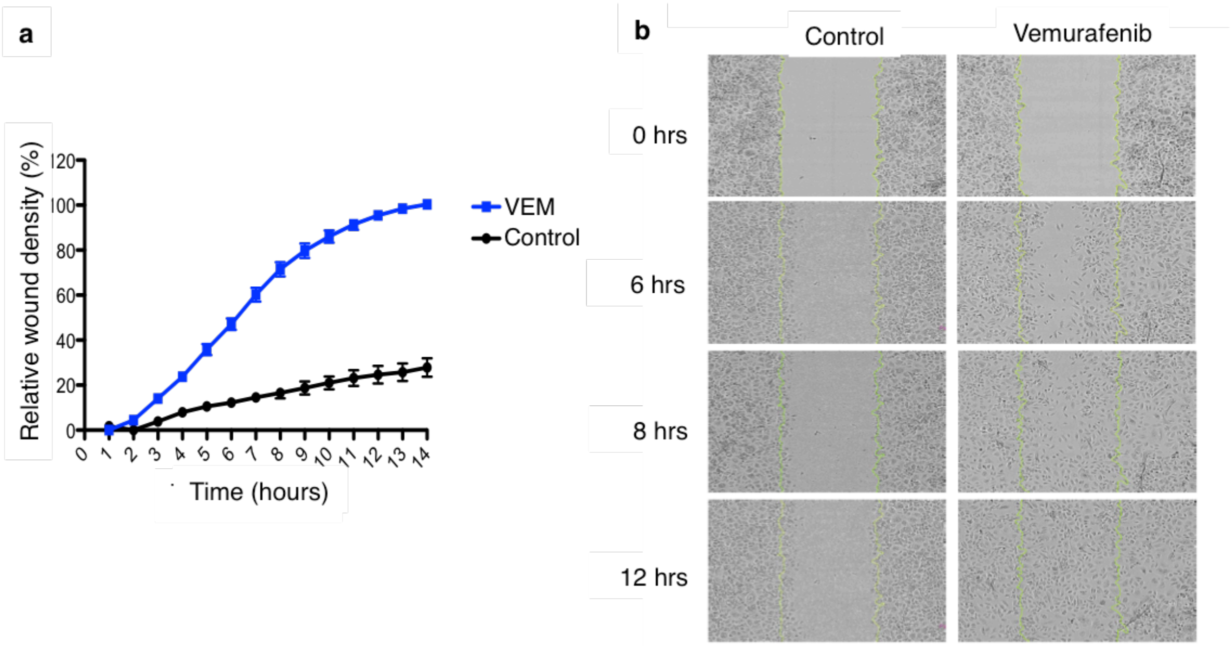

Supplementary Figure 2. BRAF inhibition in adult human epithelial keratinocytes (HEKa) increases proliferation and wound healing *in vitro*. a, Quantitative analysis of proliferation and scratch healing as the percent relative wound density of cells in the scratch area at different time points in replicate cultures of HEKa in the presence or absence of vemurafenib (VEM) by automated microscope analyzer ($p = 0.0044$ by t-test). Representative videos of the cultures are available as Supplemental Online Materials. **b**, Time-course images of cell proliferation scratch assays of HEKa in the presence or absence of vemurafenib (1.5 μM). Images and analysis done with IncuCyte Zoom Life Imaging Software. Error bars in **a**, mean ± s.d.; n=6.

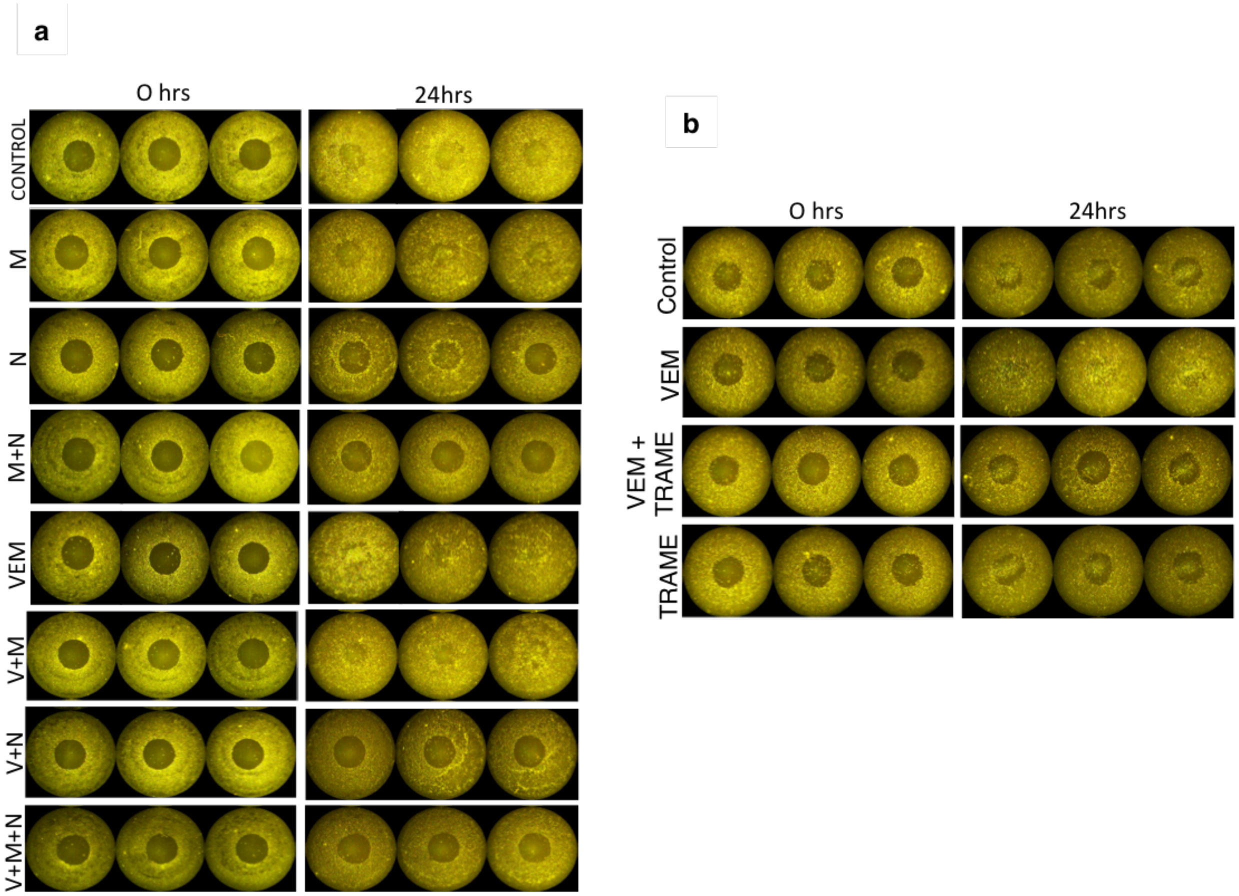

Supplementary Figure 3. BRAF inhibition induces migration in human epithelial adult keratinocytes (HEKa) leading to increased healing *in vitro*. **a**, Representative images of HEKa cell migration wound-healing assays *in vitro* in the presence or absence of mitomycin C (M, 10 μg/ml), NSC 295642 (N, 1 μg/ml), vemurafenib (VEM, 1.5 μM), or combination at 0 and 24 hours. **b**, Representative images of HEKa cell migration wound-healing assays *in vitro* in the presence or absence of vemurafenib (VEM; 1.5 μM), trametinib (TRAME; 1 μM) or the combination (VEM+TRAME) at 0 and 24 hours. Both experiments were conducted in triplicate and repeated twice.

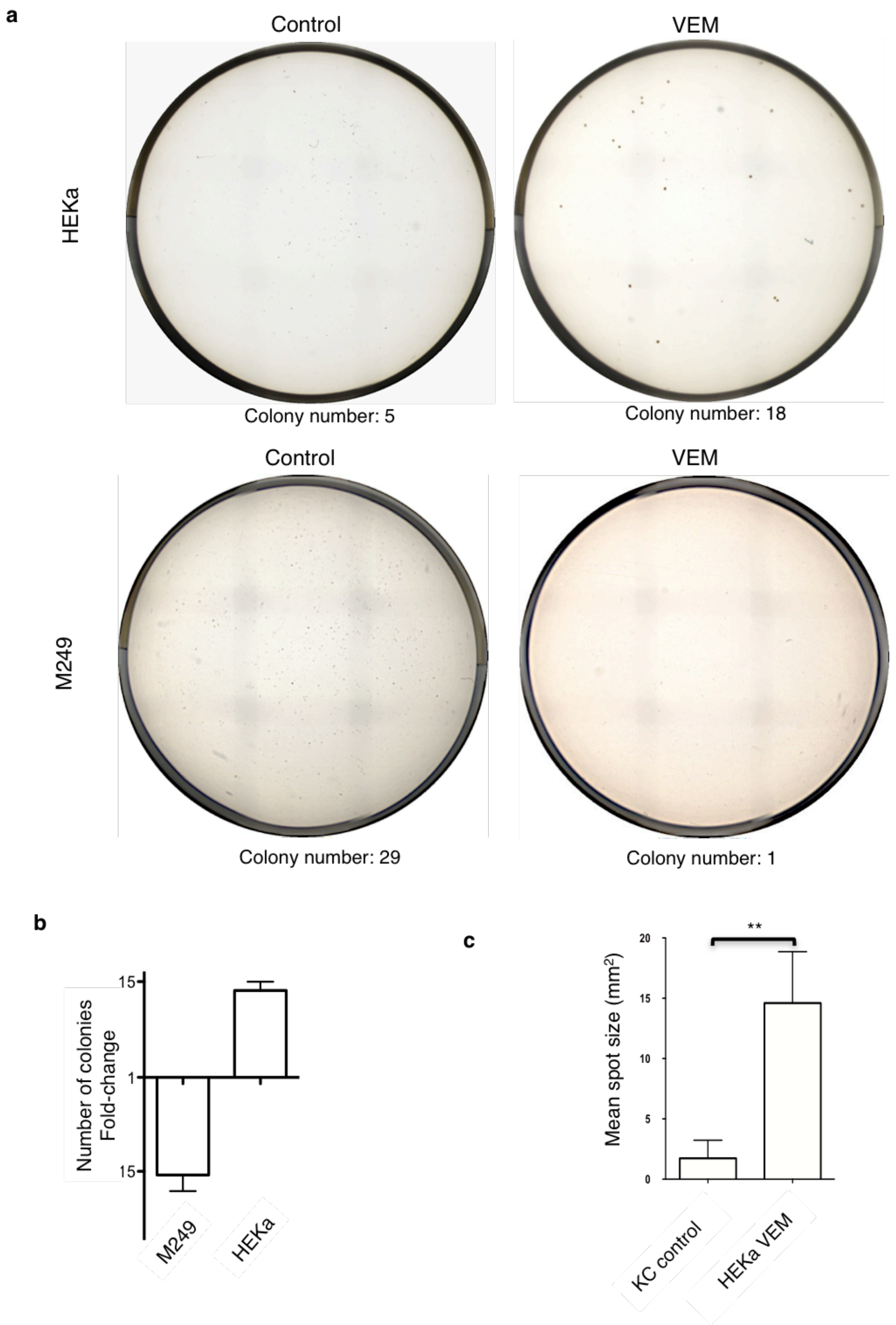

Supplementary Figure 4. BRAF inhibition induces proliferation in human epithelial adult keratinocytes (HEKa), with the opposite effect in a *BRAF*^{V600E} mutated melanoma cell line. a, Representative colony culture images of HEKa and M249 cells treated with DMSO or vemurafenib

(VEM) at 1.5 μM. Minimum gating for the colonies is 0.0025 mm^2. **b**, Fold-change of colony quantification of HEKa and the *BRAF*^{V600E} mutant melanoma cell line M249 grown in soft-agar with or without exposure to vemurafenib. **c**, Representative histogram of the mean spot size (mm^2) of HEK colonies treated with DMSO or VEM. **p = 0.007 by t-test. Error bars in **b** and **c**, mean ± s.d.; n=3.

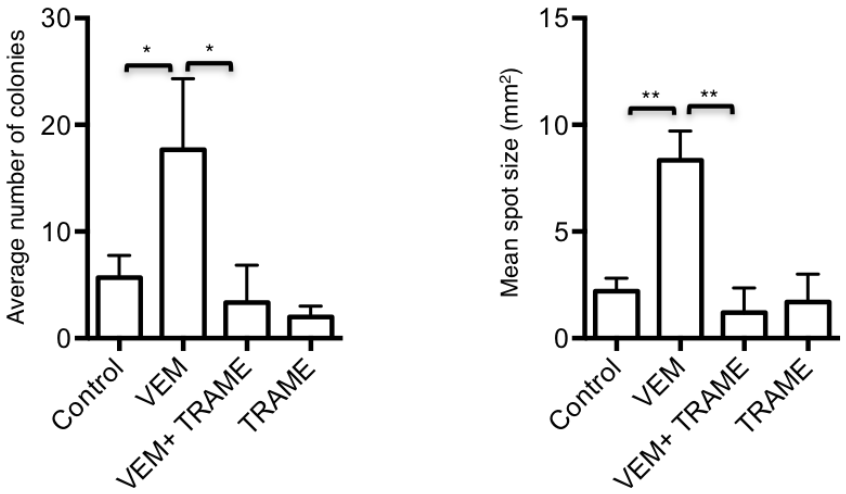

Supplementary Figure 5. Trametinib inhibits proliferation and size of HEKa colonies induced by vemurafenib. Colony number and mean spot size (mm^2) quantifications of HEKa grown in soft-agar with or without exposure to vemurafenib and/or trametinib. Minimum gating for the colonies is 0.0025 mm^2. *p = 0.04 Control vs VEM; *p = 0.03 VEM vs VEM+TRAME; **p = 0.002 Control vs VEM; **p = 0.002 VEM vs VEM+TRAME. Error bars, mean ± s.d.; n=3.

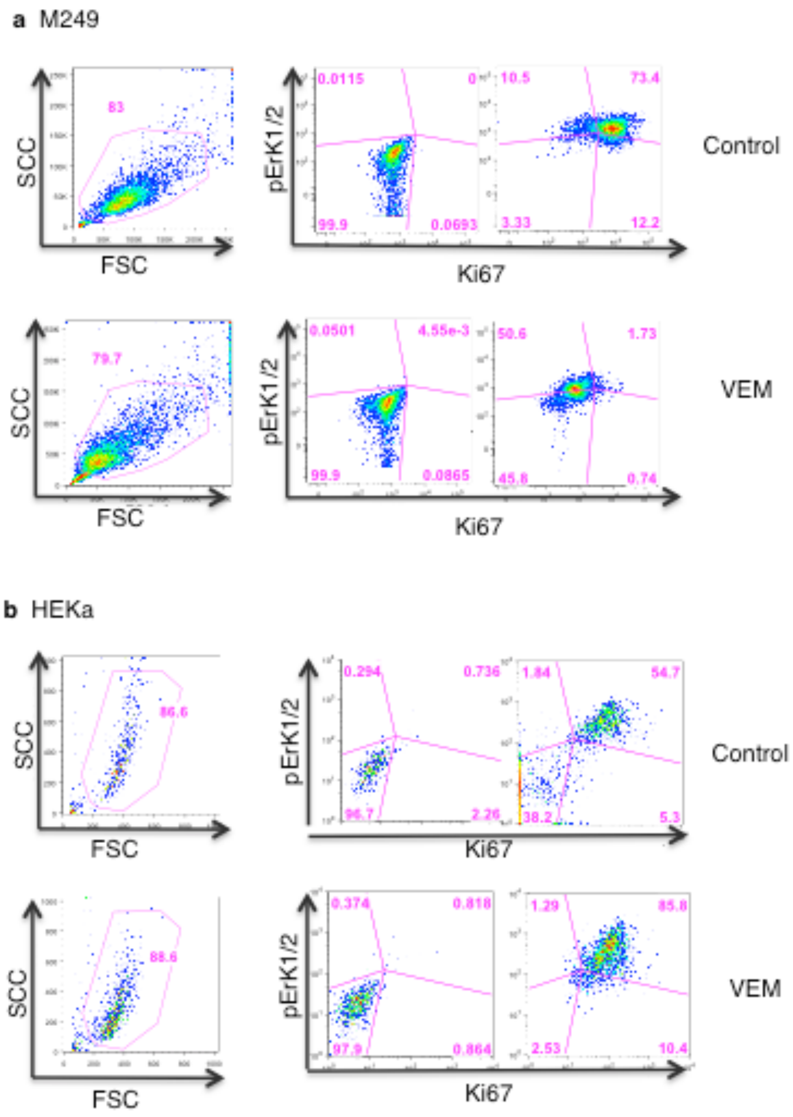

Supplementary Figure 6. Representative panel of intracellular flow cytometry analysis of HEKa and M249 cells. HEKa and M249 cells were treated with vehicle or vemurafenib (VEM) for 24 hours and co-stained with pERK and Ki67. Forward-scattered light (FSC), proportional to cell-surface size, and side-scattered light (SSC), proportional to cell granularity, are indicated on the left panels. Each co-stained cells panel (right) has its unstained control on the left.

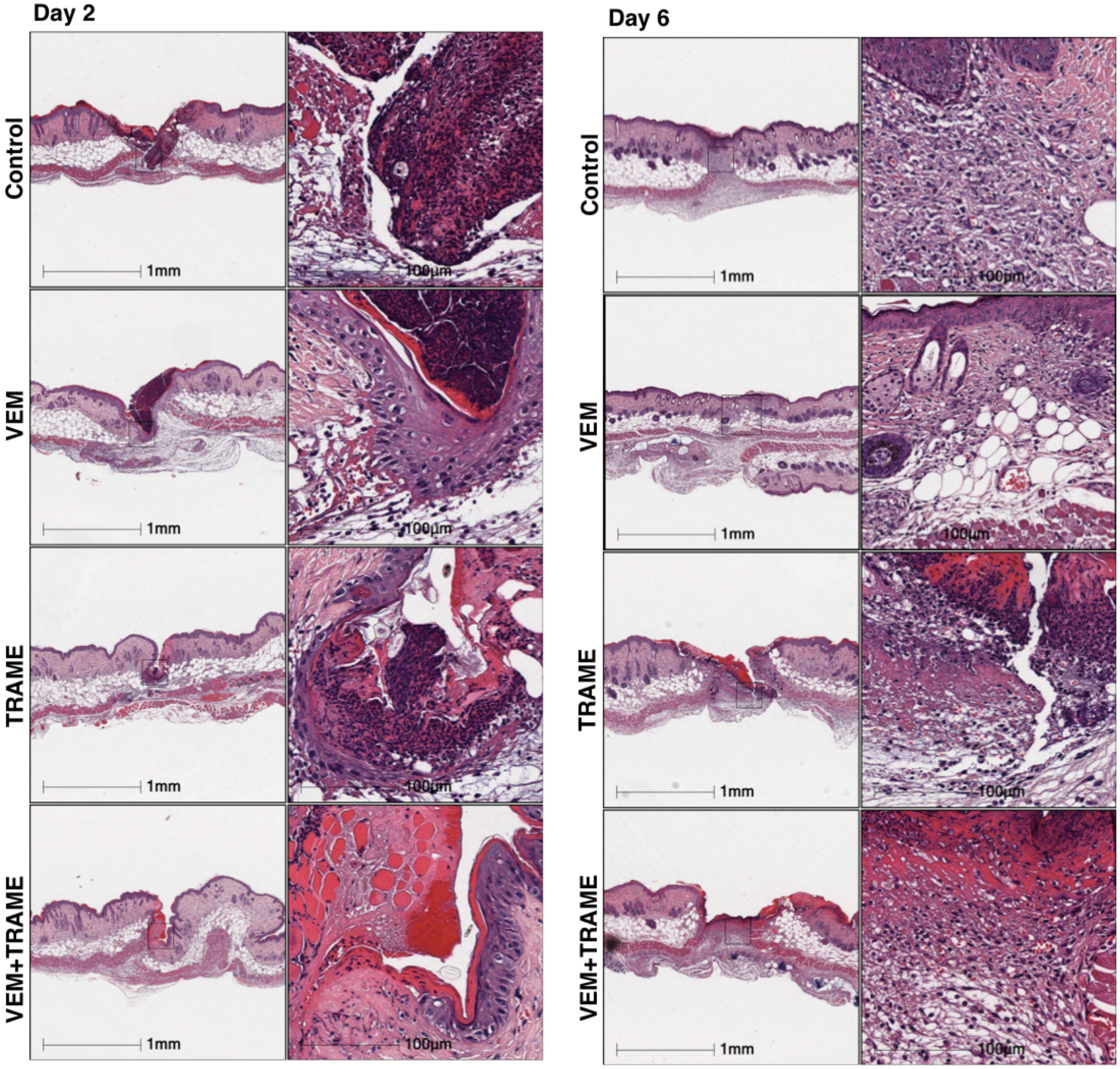

Supplementary Figure 7. **Vemurafenib accelerates and trametinib suppresses wound healing in C3H mice. a**, Representative photomicrograph hematoxylin and eosin (H&E) staining images in the presence and absence of vemurafenib (VEM), trametinib (TRAME) or the combination (VEM+TRAME) on days 2 and 6 post-treatment. In each group, the healing of incised wounds involved the same standard processes. Wound-adjacent epidermis undergoes hyperplasia and proliferation and epidermal cells from this process migrate centrally to seal the incised epidermal deficiency. The space of the incision fills initially with fibrin, which is then

colonized by fibroblasts, macrophages, polymorphonuclear cells and new capillaries. In the presence of vemurafenib the healing process is accelerated. Wound-adjacent epidermal hyperplasia was more extensive two days post-incision in the vemurafenib-treated group compared to the control specimen. Skin surface integrity is re-established in six days with beginning of sub-epidermal fibrosis (VEM panel; day 6), while, at this point, the re-epithelialization is not complete and dermal reparative fibrosis is absent in the control group (Control panel, day 6). The group treated with trametinib alone shows slight peri-lesional hyperplasia at day 2 (TRAME panel) and no evidence of repair by day 6 (TRAME panel). In the vemurafenib plus trametinib combination group (TRAME+VEM panel) peri-lesional hyperplasia is lower than in the group treated with vemurafenib alone at day 2 (VEM panel), but greater than trametinib alone. Furthermore, re-epithelialization is absent at day 6 (TRAME panel).

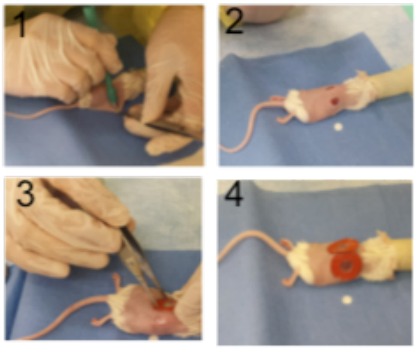

Supplementary Figure 8. Photographic representation of the excisional wound splinting model. Picture 1 shows the induced 6-mm round wound on the back of Balb/c mice. Picture 2 shows the wounds right before topical application of DMSO or vemurafenib. Pictures 3 and 4 show the adhesion of splinting rings on top of the wounds prior to suture to the skin around the wounds to prevent wound closure by skin contraction.

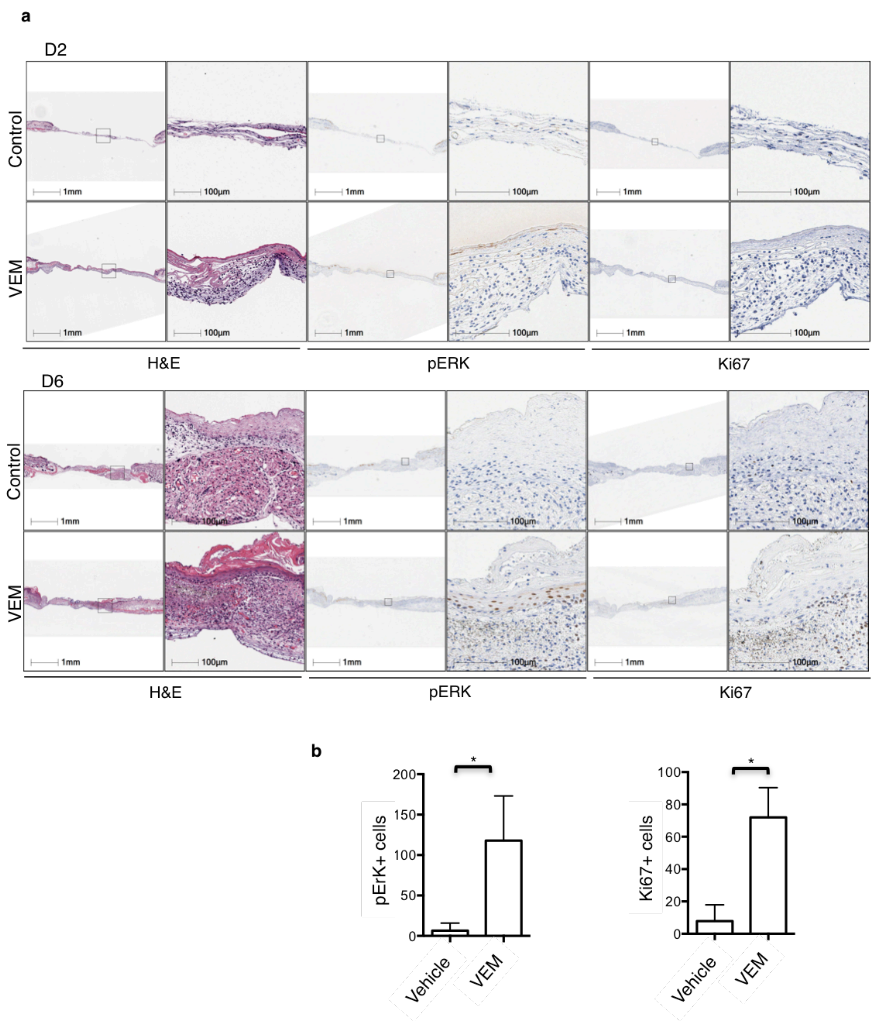

Supplementary Figure 9. Representative photomicrograph histology images in the presence and absence of vemurafenib (VEM) on day 2 and 6 post-surgery. **a,** Representative photomicrograph immunohistochemistry images on day 2 and day 6 of control and vemurafenib-treated wounds. **b,** Bar graph representing the quantification of pERK+ and Ki67+ cells on the vehicle- and vemurafenib-treated wounds on day 6 ($p = 0.02$; $n = 4$). Error bars in **b**, mean ± s.d.

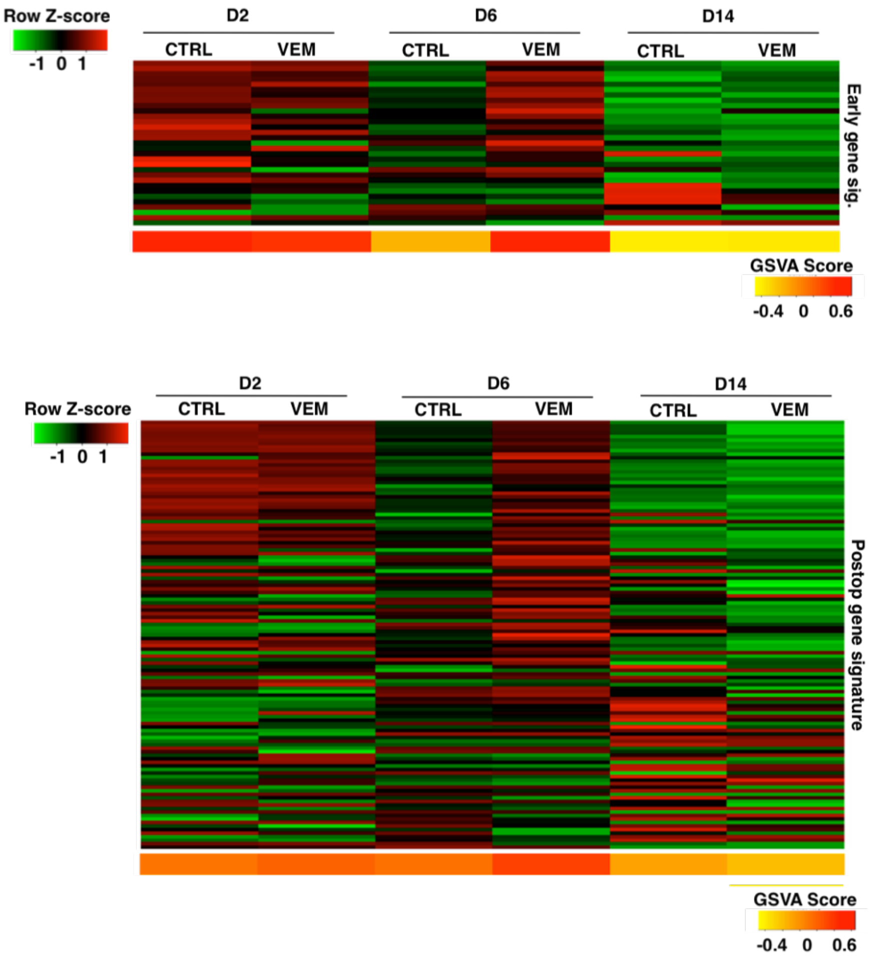

Supplementary Figure 10. Gene expression profiling of cutaneous wounds in mice with or without exposure to vemurafenib compared to a postoperative signature and an early wound healing signature. Full thickness skin resected around the areas of the initial wound was analyzed for gene expression profiling by RNASeq. **Top**, mRNA expression and overall enrichments of a post-op wound healing signature. **Bottom**, mRNA expression and overall enrichments of pro-inflammatory genes involved at the early stages of wound healing. There were enrichments of both signatures in vemurafenib-treated wounds, at day 6 compared to the control wounds. A more pronounced loss of both wound-healing signatures was observed in the vemurafenib-treated group by day 14 due to a much rapid healing process on the treated mice compared to the untreated. Genes were listed on Supplementary Table 2.

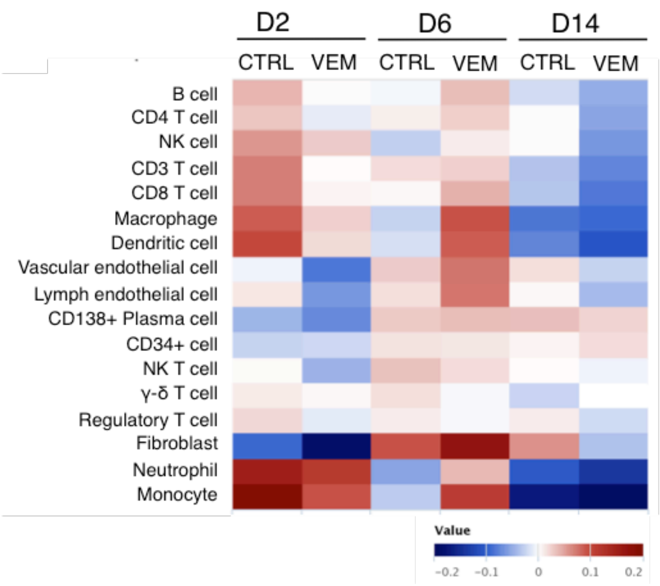

Supplementary Figure 11. BRAF inhibition activates multiple cell subsets involved in wound healing of the skin. Immune cell type-specific gene set enrichments from the RNAseq data of mice with or without exposure to vemurafenib at day 2, 6 and 14 post-treatment.

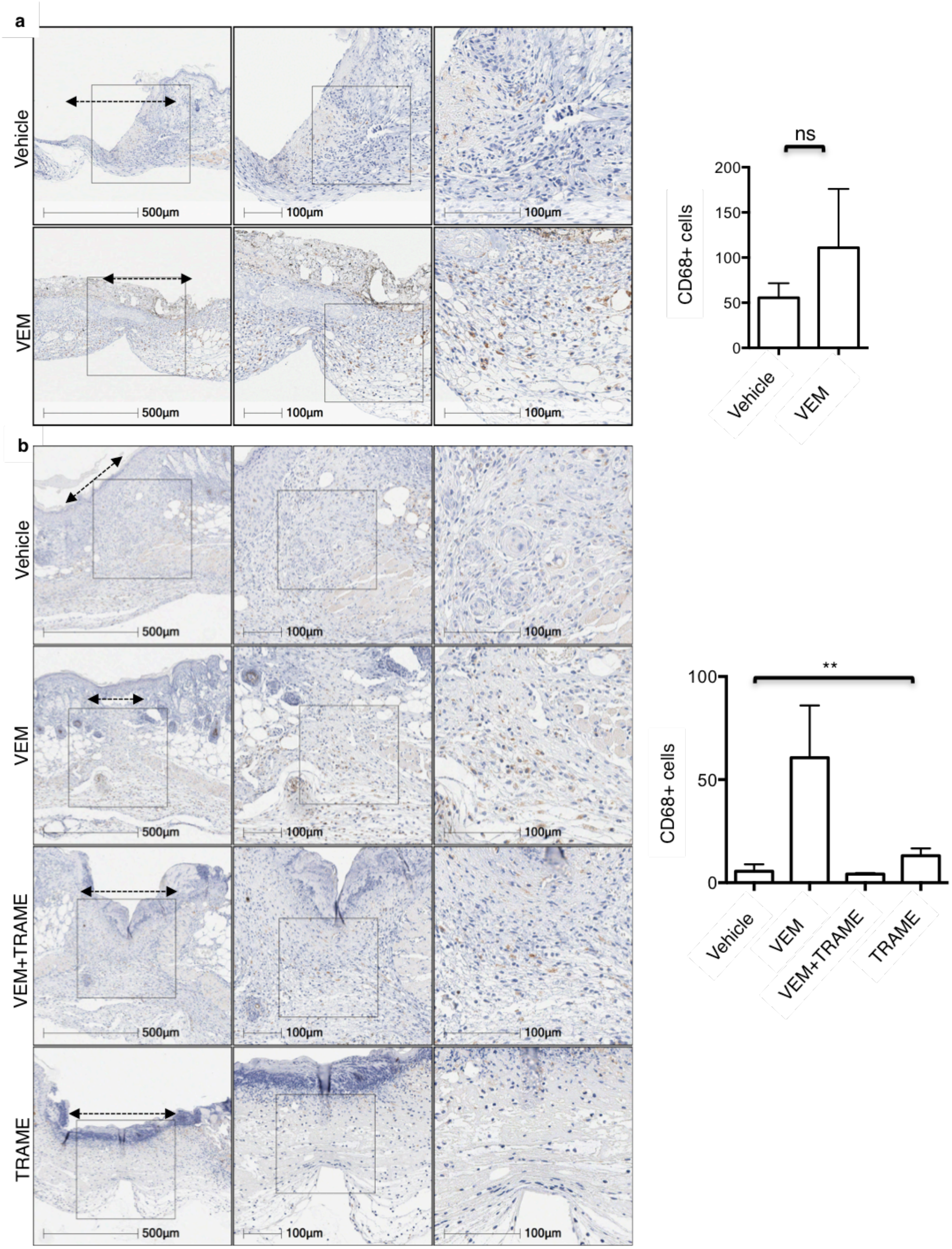

Supplementary Figure 12. Macrophages are increased in mice wounds treated with vemurafenib and reversed when adding trametinib. a, Representative photomicrograph

immunohistochemistry images of CD68+ cells in a excisional wound splinting model of mice treated with vehicle or vemurafenib on day 6 post-treatment and bar graph, on the right side, representing the quantification of CD68+ cells on the vehicle- and vemurafenib-treated wounds on day 6 ($p = 0.14$ by t-test; $n = 4$). Wound area indicated with an arrow. **b**, Representative photomicrograph immunohistochemistry images of CD68+ cells in an incisional wound model of mice treated with vehicle, vemurafenib (VEM), trametinib (TRAME) or the combination (VEM+TRAME); ($p = 0.0018$ by one-way anova; $n=4$). Wound area between double head arrows. Error bars, mean ± s.d. ns= non-significant.

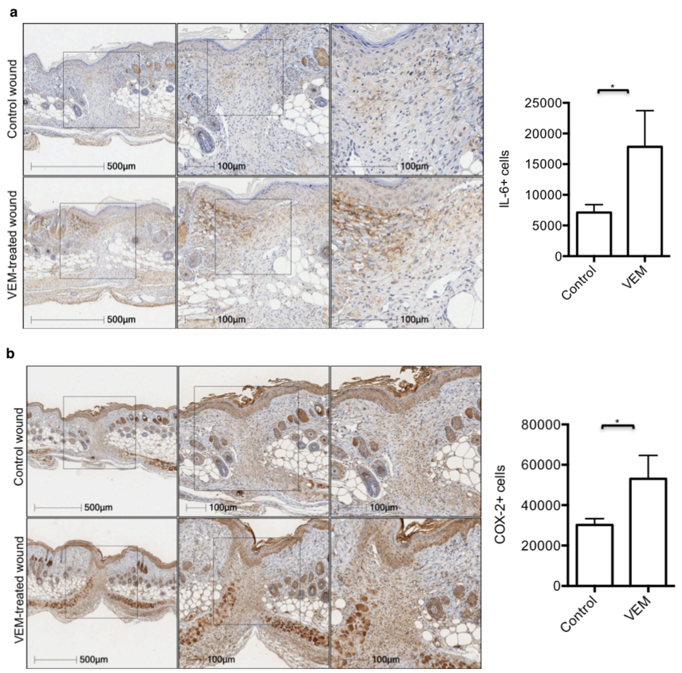

Supplementary Figure 13. Increased levels of COX-2 and IL-6 in the wound area of wounds treated with vemurafenib. a, Representative photomicrograph immunohistochemistry images of IL-6+ cells in an incisional wound model and bar graph, on the right side, representing the quantification of IL-6+ cells on the vehicle- and vemurafenib-treated wounds on day 6 ($p = 0.02$ by t-test $n=4$). **b,** Representative photomicrograph immunohistochemistry images of COX-2+ cells and bar graph, on the right side, representing the quantification of COX-2+ cells on the vehicle- and vemurafenib-treated wounds on day 6 ($p = 0.01$ by t-test; $n = 4$). Error bars, mean ± s.d.

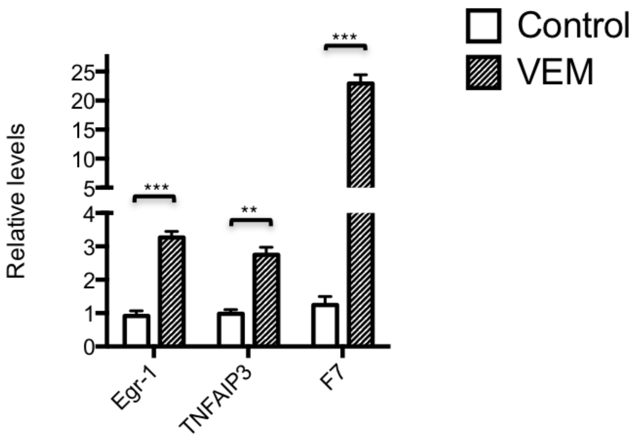

Supplementary Figure 14. Egr-1, TNFAIP3 and F7 expression levels are increased in vemurafenib-treated skin wounds. mRNA levels of Egr-1, TNFAIP3 and F7 of total skin wounds treated and untreated with vemurafenib at day 6 post-incision. Bar graphs represent experimental mean of three biological replicates and error bars represent standard error of the mean (s.e.m.). RNA levels were normalized against β-*actin.* *** p = 0.0006 (Egr-1) and 0.0002 (F7) and ** p = 0.0023 (TNFAIP3), all by t-test.

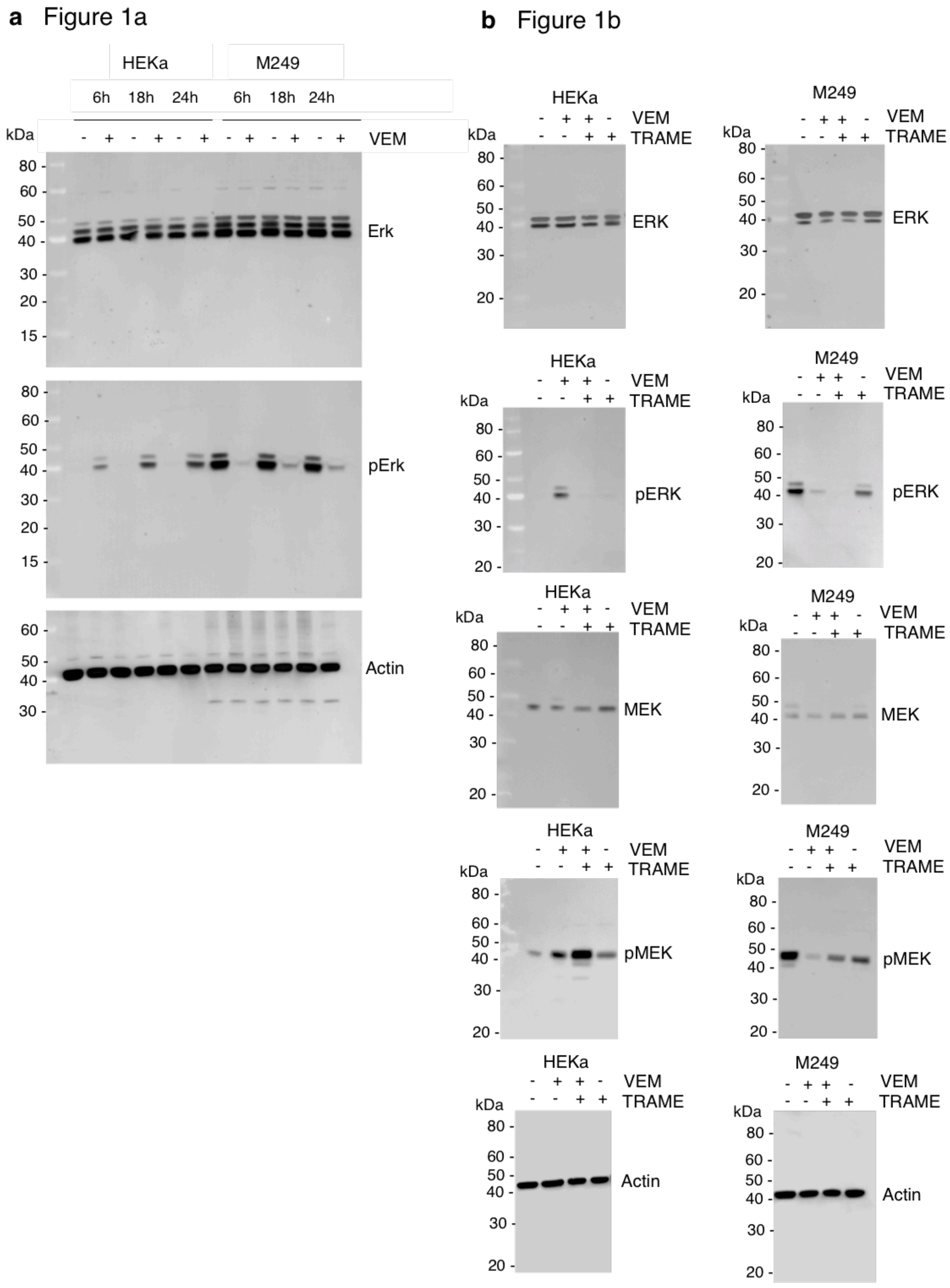

Supplementary Figure 15. Uncropped scans of the most important immunoblots.
